# ANATOMIC VARIATIONS OF THE CYSTIC ARTERY DURING CHOLECYSTECTOMIES: IS IT IMPORTANT FOR THE SURGEON TO KNOW?

**DOI:** 10.1590/0102-67202025000011e1880

**Published:** 2025-05-12

**Authors:** João Alfredo SCHIEWE, Livia Hoyer Garcia MIRANDA, Renata Marino ROMANO, Marco Aurelio ROMANO

**Affiliations:** 1 Universidade Estadual do Centro-Oeste, Department of Medicine - Guarapuava (PR), Brazil.

**Keywords:** Anatomy, Cholecystectomy, Anatomic Variation, Hepatic Artery, Anatomia, Colecistectomia, Variação Anatômica, Artéria Hepática

## Abstract

**BACKGROUND:**

Knowledge of the cystic artery and its variations is essential to perform safe cholecystectomies. The cystic artery originates from the right hepatic artery, passing posterior to the common hepatic duct, anterior to the cystic duct, and branching into two branches at the neck of the gallbladder. However, variations in position, size, and relationship with adjacent structures are common.

**AIMS:**

This article presents a literature review regarding cystic artery variations and their frequency during cholecystectomies.

**METHODS:**

The articles selected for this review were chosen from the PubMed and SciELO databases. The standardized descriptors used were anatomic variation and cholecystectomy. These were chosen using the “Medical Subject Headings” and combined with the Boolean operator AND and the non-standard descriptor cystic artery.

**RESULTS:**

It was found in 54.5% of the studies that the anatomical pattern of the cystic artery was the most frequent type. A different origin from the standard was cited in 63.6% of the articles. Double irrigation of the gallbladder was found in 59.1%. In 36.4%, the cystic artery was anterior to the common hepatic duct or the cystic duct. Cystic arteries outside Calot’s triangle were found in 36.4%. Short cystic arteries were found in 13.6%. The absence or non-identification of the artery was reported in 9.1%.

**CONCLUSIONS:**

Variations of the cystic artery are common and are frequently reported. One aspect of a safe cholecystectomy is anatomical knowledge and its possible variations. Thus, surgeons must be familiar with this point in order to reduce vascular and biliary injuries.

## RESUMO

**RACIONAL:**

O conhecimento anatômico da artéria cística e de suas variações é essencial para a realização de colecistectomias seguras. A artéria cística possui origem na artéria hepática direita, passando posteriormente ao ducto hepático comum, anteriormente ao ducto cístico, ramificando-se em dois ramos no colo da vesícula biliar. Todavia, variações de posição, tamanho e relação com estruturas adjacentes são comuns.

**OBJETIVOS:**

Este artigo apresenta uma revisão de literatura em relação às variações da artéria cística e sua frequência durante colecistectomias.

**MÉTODOS:**

Os artigos selecionados desta revisão foram escolhidos a partir das bases de dados PubMed e SciELO. Utilizou-se os descritores padronizados: Anatomic variation e cholecystectomy. Estes, foram escolhidos usando o “Medical Subject Headings” e combinados com o operador booleano AND e o descritor não padronizado Cystic artery.

**RESULTADOS:**

Verificou-se em 54,5% dos estudos que o padrão anatômico da artéria cística foi o tipo de maior ocorrência. Citou-se uma origem diferente do padrão em 63,6% dos artigos. Encontrou-se a irrigação dupla da vesícula biliar em 59,1%. Em 36,4% a artéria cística estava anteriormente ao ducto hepático comum ou ao ducto cístico. Encontrou-se artérias císticas fora do triângulo de Calot em 36,4%. Artérias císticas curtas foram encontradas em 13,6%. Relatou-se a ausência ou não identificação da artéria em 9,1%.

**CONCLUSÕES:**

Variações da artéria cística são comuns, sendo frequentemente relatadas. Um aspecto de uma colecistectomia segura é o conhecimento anatômico e de possíveis variações. Assim, cirurgiões devem estar familiarizados com este ponto a fim de reduzir lesões vasculares e biliares.

**Figure 1:**
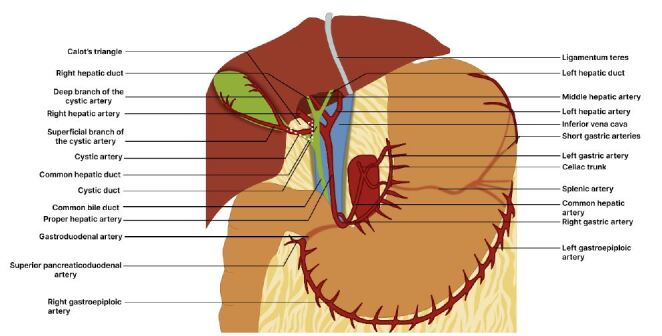
Boundaries of Calot’s triangle and its contents, as well as the normal course of the cystic artery to the gallbladder. Additionally, some arteries from which the cystic artery may have a variant origin are also presented (Author’s Source).

**Central Message**

First described by Jean-François Calot in 1891, the hepatobiliary triangle is an anatomical landmark of undeniable importance for performing cholecystectomies. The cystic artery supplies the gallbladder and originates from the right hepatic artery, passing posterior to the common hepatic duct, anterior to the cystic duct, and reaching the upper part of the gallbladder neck, where it divides into two branches: a superficial branch, which runs along the peritoneal surface of the gallbladder, and a deep branch, which lies in the gallbladder fossa between the gallbladder and the liver. Its position, size, and relationship with adjacent structures influence surgical procedures for a safe cholecystectomy, as its origin, number, and course can vary, as well as its presence within the triangle.

**Perspectives**

Variations of the cystic artery are not uncommon findings, with different anatomical variations being reported during cholecystectomies, as cited in the present review. One of the key aspects of a safe cholecystectomy is knowledge of the anatomy and potential anatomical variations. Therefore, surgeons should be familiar with these aspects during cholecystectomies to reduce the incidence of vascular or biliary injuries. Thus, the fundamental importance of understanding the possible variations of this structure is emphasized, as it may have implications for surgical interventions and imaging studies related to the abdominal region.

## INTRODUCTION

First described by Jean-François Calot in 1891, the hepatobiliary triangle is an anatomical landmark of undeniable importance for performing cholecystectomies^1^. Fundamentally, this triangle is bounded superiorly by the inferior border of the liver, inferiorly by the cystic duct, and medially by the common hepatic duct (Figure 1). Its contents include the cystic artery, a variable portion of the right hepatic artery, the cystic lymph node and lymphatic vessels, as well as fibrous-adipose connective tissue^1^ ^,^ ^13^ ^,^ ^20^.

**Figure 1:**
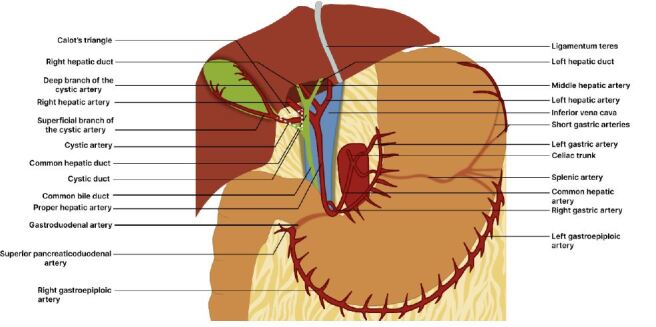
Boundaries of Calot’s triangle and its contents, as well as the normal course of the cystic artery to the gallbladder. Additionally, some arteries from which the cystic artery may have a variant origin are also presented (Author’s Source).

The cystohepatic triangle is an area where anatomical variations are commonly found and can impact surgical performance during cholecystectomy. It is unquestionable that, in addition to surgical skills and techniques, knowledge and understanding of Calot’s triangle enable digestive system surgeons to manage potential anatomical variations and carefully dissect the triangle to properly identify the region and avoid injury to the extrahepatic biliary tree and blood vessels, as cystic artery bleeding is a significant complication during cholecystectomies due to its impact on abdominal visibility^11^ ^,^ ^12^.

The cystic artery supplies the gallbladder and originates from the right hepatic artery, passing posterior to the common hepatic duct, anterior to the cystic duct, and reaching the upper part of the gallbladder neck, where it divides into two branches: a superficial branch, which runs along the peritoneal surface of the gallbladder, and a deep branch, which lies in the gallbladder fossa between the gallbladder and the liver (Figure 1)^1^ ^,^ ^8^. Its position, size, and relationship with adjacent structures influence surgical procedures for a safe cholecystectomy, as its origin, number, and course may vary, as well as its presence within the triangle^6^ ^,^ ^13^.

The anatomical knowledge of Calot’s triangle is considered an important factor in reducing surgical complications^5^ ^,^ ^9^. Additionally, it allows for better planning and interventions in unique cases. For these reasons, the search for records on anatomical variations of the cystic artery found during cholecystectomies was necessary, as well as their clinical implications.

## METHODS

This study was conducted through a systematic literature review with a qualitative exploratory approach from July to October 2022^7^. The articles used for this review were selected from the PubMed and SciELO databases. For the development of this review, a preliminary research phase was carried out, involving an extensive investigation of the anatomical variations of the cystic artery during cholecystectomies, leading to the research question: “What are the prevalence rates of anatomical variations of the cystic artery found during cholecystectomies?” Studies related to the topic were selected and read in full, with those unrelated to the study being excluded. The studies that were relevant to the research were discussed, and their results are presented in this review. For the selection of these studies, standardized descriptors were chosen using MeSH (Medical Subject Headings) and combined with the Boolean operator AND, along with the non-standardized descriptor cystic artery. The terms used in the study search are presented in Table 1.

**Table 1:** Search terms used in database research.

Database	Search terms
SciELO	cystic artery AND anatomic variation AND cholecystectomy
PubMed	cystic artery AND anatomic variation AND cholecystectomy

For data analysis, exclusion and inclusion criteria were established, which were assessed by two reviewers guided by the research question and eligibility criteria. The inclusion method focused on studies describing variations of the cystic artery based on cholecystectomies performed on humans over 18 years of age. Thus, full articles and case reports written in English or Spanish were included, regardless of the year of publication. As for the exclusion criteria, review articles, studies conducted on animal models, data obtained from cadaveric dissections and tomography, research involving human samples under 18 years of age, duplicate articles, or studies whose content did not align with the review’s objective were not selected.

## RESULTS

**Figure 2:**
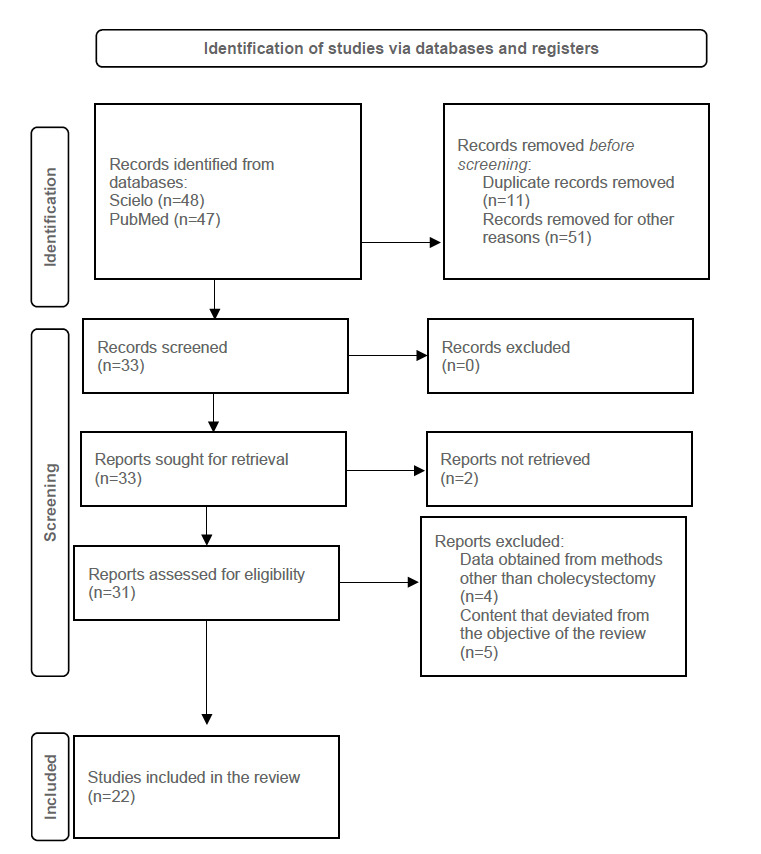
Search and selection of studies for the systematic review according to PRISMA guidelines.

The study identification diagram based on database searches and PRISMA guidelines is presented in Figure 2. Initially, 95 articles were identified through database searches using standardized descriptors. Of these, 62 were excluded for lacking relevant data or being duplicates across platforms. During the screening stage, 33 studies were selected, with none excluded after title and abstract analysis. These studies were then selected for full-text reading, and 2 were excluded due to unavailability in full. Thus, 31 articles were evaluated based on the eligibility criteria, and of these, 22 were included in the final analysis sample.

Table 2 presents the main findings of the studies used for discussion. It is stratified by year of publication, study sample, and main results.

**Table 2:** Characteristics of studies evaluating the occurrence of anatomical variations of the cystic artery in humans during cholecystectomies.

Author	Study sample	Main results
Ding et al.^9^	600 patients (232 men and 368 women)	In 440 patients (73.3%), the cystic artery passed through Calot’s triangle, originated from the right hepatic artery, and was single. In 73 patients (12.2%), the artery passed through Calot’s triangle, originated from the right hepatic artery, and was double, with an anterior and a posterior branch. In 45 patients (7.5%), the artery did not pass through Calot’s triangle and originated from the gastroduodenal artery. In 18 patients (3.0%), the artery did not pass through Calot’s triangle and originated from variants of the right hepatic artery. In 15 patients (2.5%), the artery did not pass through Calot’s triangle and originated directly from the liver parenchyma. In nine patients (1.5%), there was more than one artery supplying the gallbladder: 5 (0.8%) had a normal cystic artery within Calot’s triangle with an additional artery posteriorly crossing the cystic duct and some small arteries extending directly from the liver parenchyma to the gallbladder; 3 (0.5%) had an additional cystic artery (besides the normal one passing through Calot’s triangle) superficial to the cystic duct; and 1 (0.2%) had multiple cystic arteries, one double artery passing through the triangle, one crossing the common bile duct anteriorly, and another situated on the right side of the gallbladder’s body and fundus edge.
Fateh et al.^11^	1,850 patients (1658 women and 192 men)	In 1,752 patients (94.7%), the cystic artery was superomedial to the cystic duct. In 10 patients (0.5%), the cystic artery was posteromedial to the cystic duct. In 5 patients (0.3%), the cystic artery was anterior to the cystic duct. Three patients (0.2%) did not have a cystic artery.
Kim et al.^16^	Single (1 male patient)	Double cystic artery, one originating from the right hepatic artery and the other from the hepatic artery of liver segment IV.
Talpur et al.^28^	300 patients (255 women and 45 men)	Among the 300 patients, 32 (10.7%) had abnormalities of the cystic artery, as follows: 8 (2.7%) cystic arteries anterior to the cystic duct; 7 (2.3%) with origins different from the right hepatic artery; 5 (1.7%) short cystic arteries; 4 (1.3%) cystic arteries posterior to the cystic duct; 3 (1%) cystic arteries arising above Calot’s triangle; 3 (1%) double cystic arteries; and 2 (0.7%) cystic arteries to the right of the cystic duct.
Kim and Yoon^15^	Single (1 male patient)	Cystic artery originating from the middle hepatic artery.
Suzuki et al.^27^	244 patients (unspecified sex)	In 187 patients (76.7%), a single cystic artery passing through Calot’s triangle was found. In 27 patients (11.1%), no artery was present in Calot’s triangle. In 13 patients (5.3%), there was a double blood supply to the gallbladder, with a single cystic artery passing through Calot’s triangle and an accessory artery located inferolateral to the cystic duct. In 6 patients (2.5%), the cystic artery was single, passed through Calot’s triangle, and during its course, “hooked” around the cystic duct posteriorly, reappearing on the peritoneal surface near the gallbladder neck. In 6 patients (2.5%), a double cystic artery passing through Calot’s triangle was found. In 3 patients (1.2%), there was a double blood supply to the gallbladder, with a single cystic artery passing through Calot’s triangle and the accessory artery perforating the gallbladder bed. In 1 patient (0.4%), there was a double blood supply to the gallbladder, with a single cystic artery passing through Calot’s triangle and the accessory artery running along with the cystic duct. In 1 patient (0.4%), there was a double blood supply to the gallbladder, with a single cystic artery passing through Calot’s triangle and the accessory artery located just below Hartmann’s pouch.
Martín Pérez et al.^18^	Single (1 female patient)	Short and single cystic artery originating from the “Moynihan’s hump” (an abnormal variation of the right hepatic artery).
Nguyen et al.^21^	Single (1 female patient)	Cystic artery passing anterior to the common bile duct from right to left toward the gallbladder on the left side without situs viscerum inversus.
Pavlidis et al.^23^	Single (1 female patient)	Presence of additional branches of the cystic artery, such as a posterior branch coursing toward the undersurface of the gallbladder in a patient with previously unknown total situs inversus.
Yamazaki et al.^30^	Single (1 male patient)	Cystic artery was found on the left side of the gallbladder, originating from an aberrant artery that passed anteriorly to the fundus of the gallbladder and coursed toward hepatic segment V. The aberrant artery arose from the left hepatic artery.
Balija et al.^4^	200 patients (unspecified sex)	In 189 patients (94.5%), the cystic artery passed through the hepatobiliary triangle, as follows: 147 (73.5%) normal cystic arteries; 31 (15.5%) double cystic arteries, with one anterior and one posterior branch; and 11 (5.5%) cystic arteries originating from an aberrant right hepatic artery. In 11 patients (5.5%), the cystic artery did not pass through the hepatobiliary triangle, as follows: 9 (4.5%) cystic arteries originating from the gastroduodenal artery and 2 (1%) originating from the left hepatic artery.
Singh et al.^26^	740 patients (280 men and 460 women)	In 340 patients (45.9%), there was a single cystic artery. In 240 patients (32.4%), there were two cystic arteries, one superficial and one deep. In 28 patients (3.8%), there was a single short cystic artery originating from the right hepatic artery within Calot’s triangle. In 17 patients (2.3%), there was a single short cystic artery not originating from the right hepatic artery, crossing the common hepatic duct anteriorly. In 23 patients (3.1%), neovascularization was noted, with the presence of small vessels in the areolar and adipose tissues. In 27 patients (3.6%), there was a double or accessory cystic artery. In 15 patients (2%), there were vessels originating directly from the liver. In 47 patients (6.4%), there was a vessel posterolateral to the margin of the gallbladder. In 22 patients (3%), the cystic arteries were observed more anteriorly than posteriorly in relation to the lymph node. In 18 patients (2.4%), the cystic artery initially crossed the cystic duct laterally and terminated medially to the cystic duct.
Nagendram et al.^19^	Single (1 female patient)	Cystic artery crossing anteriorly to the cystic duct from right to left in a patient with a left-sided gallbladder without total situs inversus.
Katagiri et al.^14^	Single (1 male patient)	The anterior cystic artery adhered to the cystic duct outside Calot’s triangle, originating from an aberrant right hepatic artery arising directly from the celiac trunk.
Pereira-Graterol et al.^24^	Single (1 female patient)	The cystic artery located along the posterior wall of the body of the gallbladder, not identified during the initial dissection of Calot’s triangle in a patient with total situs inversus.
Torres et al.^29^	84 patients (16 men and 64 women)	In 60.7% of patients, the cystic artery originated from the right hepatic artery. In 7.2% of patients, the cystic artery originated from the common hepatic artery, its bifurcation, or the left hepatic artery. In 6% of patients, the cystic artery originated from the gastroduodenal artery. In 26.2% of patients, the cystic artery reached the gallbladder at the fundus or the distal part of the body as a recurrent artery.
Zubair et al.^31^	220 patients (unspecified sex)	In 192 patients (87.3%), the cystic artery was observed within Calot’s triangle, of which 166 (75.5%) were single, 26 (11.8%) were double, 4 (1.8%) had the cystic artery syndrome, and 2 (0.9%) originated from an aberrant right hepatic artery. In 12 patients (5.5%), more than one blood vessel was observed, with one inside Calot’s triangle and the other outside, of which 4 (1.8%) had the vessel outside the triangle passing caudally and parallel to the cystic duct, and 8 (3.6%) had the vessel outside the triangle passing between the gallbladder and the hepatic parenchyma along the right lateral border of the gallbladder, giving off multiple small branches to this organ. Additionally, in 16 patients (7.3%), cystic arteries were observed only outside Calot’s triangle, of which 14 (6.4%) had a single artery and 2 (0.9%) had multiple vessels.
Eken et al.^10^	Single (1 female patient)	Double cystic artery originating from the gastroduodenal artery.
Singh et al.^25^	600 patients (unspecified sex)	In 426 patients (71%), single cystic arteries were found within Calot’s triangle. In 88 patients (14.7%), double cystic arteries were found within Calot’s triangle. In 42 patients (7%), cystic arteries were found outside Calot’s triangle, originating from the gastroduodenal artery. In 28 patients (4.7%), cystic arteries were found outside Calot’s triangle, originating from a variant of the right hepatic artery running parallel to the cystic duct. In 10 patients (1.7%), cystic arteries were found outside Calot’s triangle, originating from the hepatic parenchyma. In 6 patients (1%), there was blood supply to the gallbladder both outside and inside Calot’s triangle.
Larobina et al.^17^	186 patients (unspecified sex)	In 164 patients (88.2%), the cystic artery was considered normal. In 16 patients (8.6%), the cystic artery was anterior to the cystic duct. In 4 patients (2.2%), the cystic artery was located directly on top of the cystic duct. In 2 patients (1.1%), the cystic artery was not identified. In 6 patients (3.2%), there was a double cystic artery, among which, in 3 (1.6%), there were anterior and posterior branches, and in 3 (1.6%), both arteries were posterior to the cystic duct.
Akay and Leblebici^2^	360 patients (76 men and 284 women)	In 11 patients (3.1%), double cystic arteries were found, comprising 2 men and 9 women.
Noguera et al.^22^	2,000 patients (unspecified sex)	In 1,831 patients (91.6%), a single cystic artery was found within Calot’s triangle, with an apparent origin from the right hepatic artery. In 96 patients (4.8%), a double cystic artery was observed, with one inside Calot’s triangle and the other outside it (accessory cystic artery). In 44 patients (2.2%), the cystic artery was located outside the triangle, originating from the common hepatic artery. In 22 patients (1.1%), the cystic artery was within the triangle, crossing the anterior surface of the common bile duct to run transversely above the cystic duct, with a possible origin from the common hepatic artery. In 6 patients (0.3%), a double cystic artery was observed, with one at the upper border of Calot’s triangle attached to the hepatic margin and the other external to the cystic duct, coursing downward and inward, crossing the common bile duct below the liver, with a possible origin from the gastroduodenal artery.
Zubair et al.^31^	220 patients (unspecified sex)	In 192 patients (87.3%), the cystic artery was observed within Calot’s triangle, of which 166 (75.5%) were single, 26 (11.8%) were double, 4 (1.8%) had the cystic artery syndrome, and 2 (0.9%) originated from an aberrant right hepatic artery. In 12 patients (5.5%), more than one blood vessel was observed, with one inside Calot’s triangle and the other outside, of which 4 (1.8%) had the vessel outside the triangle passing caudally and parallel to the cystic duct, and 8 (3.6%) had the vessel outside the triangle passing between the gallbladder and the hepatic parenchyma along the right lateral border of the gallbladder, giving off multiple small branches to this organ. Additionally, in 16 patients (7.3%), cystic arteries were observed only outside Calot’s triangle, of which 14 (6.4%) had a single artery and 2 (0.9%) had multiple vessels.
Eken et al.^10^	Single (1 female patient)	Double cystic artery originating from the gastroduodenal artery.
Singh et al.^25^	600 patients (unspecified sex)	In 426 patients (71%), single cystic arteries were found within Calot’s triangle. In 88 patients (14.7%), double cystic arteries were found within Calot’s triangle. In 42 patients (7%), cystic arteries were found outside Calot’s triangle, originating from the gastroduodenal artery. In 28 patients (4.7%), cystic arteries were found outside Calot’s triangle, originating from a variant of the right hepatic artery running parallel to the cystic duct. In 10 patients (1.7%), cystic arteries were found outside Calot’s triangle, originating from the hepatic parenchyma. In 6 patients (1%), there was blood supply to the gallbladder both outside and inside Calot’s triangle.
Larobina et al.^17^	186 patients (unspecified sex)	In 164 patients (88.2%), the cystic artery was considered normal. In 16 patients (8.6%), the cystic artery was anterior to the cystic duct. In 4 patients (2.2%), the cystic artery was located directly on top of the cystic duct. In 2 patients (1.1%), the cystic artery was not identified. In 6 patients (3.2%), there was a double cystic artery, among which, in 3 (1.6%), there were anterior and posterior branches, and in 3 (1.6%), both arteries were posterior to the cystic duct.
Akay and Leblebici^2^	360 patients (76 men and 284 women)	In 11 patients (3.1%), double cystic arteries were found, comprising 2 men and 9 women.
Noguera et al.^22^	2,000 patients (unspecified sex)	In 1,831 patients (91.6%), a single cystic artery was found within Calot’s triangle, with an apparent origin from the right hepatic artery. In 96 patients (4.8%), a double cystic artery was observed, with one inside Calot’s triangle and the other outside it (accessory cystic artery). In 44 patients (2.2%), the cystic artery was located outside the triangle, originating from the common hepatic artery. In 22 patients (1.1%), the cystic artery was within the triangle, crossing the anterior surface of the common bile duct to run transversely above the cystic duct, with a possible origin from the common hepatic artery. In 6 patients (0.3%), a double cystic artery was observed, with one at the upper border of Calot’s triangle attached to the hepatic margin and the other external to the cystic duct, coursing downward and inward, crossing the common bile duct below the liver, with a possible origin from the gastroduodenal artery.

## DISCUSSION

This review aimed to investigate the variant forms of the cystic artery described through the analysis of cholecystectomies. In most of the included studies (54.5%), it was found that the normal anatomical pattern of the cystic artery was the most frequently occurring type. This is the expected pattern for the majority of individuals. The cystic artery arises from the right hepatic artery and courses within Calot’s triangle to the right and posterior to the common hepatic duct, then passes superiorly to the cystic duct at the neck of the gallbladder, bifurcating into a superficial and a deep branch to supply the gallbladder and the cystic duct^3^.

In the study by Noguera et al.^22^, it was observed that 91.6% of patients exhibited the classic presentation pattern of the cystic artery. Similarly, Larobina et al.^17^ observed this pattern in 88.2% of patients. In the study by Singh et al.^25^, the same pattern was noted in 71% of patients.

Cystic arteries with origins different from the classic pattern (right hepatic artery) were reported in 14 of the 22 articles selected in this review (63.6%). Among these 14 articles, 6 reported cystic arteries originating from the gastroduodenal artery, 6 from variants of the right hepatic artery, 2 from the left hepatic artery, 2 from the common hepatic artery or its bifurcation, 1 from the middle hepatic artery, 1 from the hepatic segment IV artery, 1 from the “Moynihan’s hump,” and in 2, the abnormal origin was not specified^16^. Additionally, 3 studies reported cystic arteries originating directly from the hepatic parenchyma.

Dual blood supply to the gallbladder was found in 59.1% of the selected articles. Among these, the most common presentation pattern was a superficial or anterior cystic artery and a deep or posterior one.

Variations were also observed regarding the position of the cystic artery in relation to the components of the biliary tree. In 8 of the 22 articles (36.4%), the cystic artery was located anteriorly to the common hepatic duct or the cystic duct.

Another finding present in the studies included in this review was the presence of cystic arteries outside Calot’s triangle, a variation reported in 36.4% of the selected articles.

Other described variations include the presence of short cystic arteries, mentioned in 3 of the 22 articles (13.6%) selected for this review. Finally, the absence or non-identification of the cystic artery was reported in 2 articles (9.1%).

## CONCLUSIONS

Variations of the cystic artery are not uncommon findings, with different anatomical variations being reported during cholecystectomies, as cited in this review. One of the key aspects of a safe cholecystectomy is the understanding of anatomy and possible anatomical variations. Therefore, surgeons must be familiar with these aspects during cholecystectomies to reduce the incidence of vascular or biliary injuries. Thus, the fundamental importance of understanding the possible variations of this structure is emphasized, as it may have implications for surgical interventions and imaging studies related to the abdominal region.
